# Some Considerations on the Applications of Physiology to Medicine

**Published:** 1920-03

**Authors:** G. A. Buckmaster

**Affiliations:** Professor of Physiology in the University of Bristol


### SOME CONSIDERATIONS ON THE APPLICATIONS OF PHYSIOLOGY TO MEDICINE.

BY

G. A. BUCKMASTER,

*Professor of Physiology in the University of Bristol.*

KNOWLEDGE has been likened by Montaigne to the settling of wine. The sediment collected at the bottom represents that which has separated out as accepted truth from that which is in process by clarifying, while the scum at the top represents that doubtful stuff which may never be of any use at all. This simile is peculiarly applicable to the process which is at work in those subjects termed by W. K. Clifford the inexact as contrasted with the exact sciences. Among the former physiology would undoubtedly be included.

For many years I have held that just as there are applications of chemistry or mathematics to technical subjects, so have I believed that this was possible for physiology. Responsible for the introduction of the term applied physiology, by which I understand the physiological behaviour of human beings which must stand in some sort of relation to pathology and medicine, if this science is really to justify the demands it makes on the student, I have always maintained that the teaching of human physiology should be entrusted to those with a sufficiently catholic taste to realise that, although the phenomena of life in the widest sense are exhibited by all living organisms, a branch of physiology exists in which our interests are more centred upon the exhibition of these phenomena by the human being than by the sea-anemone, earthworm or frog.

Simple explanations are often preferable to wild theories as to the causation of phenomena, and this is particularly well seen when our attention is given to the factors which influence the interchange of fluid between blood and tissue spaces. The work of F. H. Scott affords a complete explanation of the undoubted rise in hæmoglobin and corpuscular content of the blood at high levels.

More than twenty-five years ago, in 1893 and 1894, C. T. Dent, C. Slater and myself during the summer months attempted to decide whether the polycythæmia which occurs at high levels is real or apparent. Our observations were made during residence at various places of known elevation above the sea-level, one of which necessitated a stay for five days in the Vallot hut on Mont Blanc (14,600 feet). It is unnecessary to describe our work in full detail, beyond stating that all the blood-counts were made in the following manner. Paired counting chambers were used for each determination. A whole field of the microscope containing 64 squares was photographed so that the count could be made at leisure. Observations were rejected where the paired counts varied more than 10 per cent. We found that the polycythæmia by high levels is established rapidly, indeed within twelve hours. Both the corpuscles and hæmoglobin contents augment in the same degree. On descent to the sea-level, the corpuscular content and hæmoglobin per centage fall almost to the normal value within thirty-six hours.

A consideration of F. H. Scott's paper explains, I believe, this apparent increase of corpuscles and hæmoglobin-content at high levels. He has completely disposed of two statements which are met with not infrequently. He finds that the hæmoglobin content of the blood in the capillaries is identical with that in the blood of the carotid artery, and his experimental evidence is conclusive in establishing the fact that the body does not contain masses of corpuscles stored away in various organs, such as the spleen or liver, which are liberated into the general circulation in times of stress, such as would occur, for example, after hemorrhage. He shows that the essential factor which causes an interchange of fluid between the blood and tissue-spaces is the arterial blood-pressure.

In all experiments, such as those in which the animal struggled, the early stages of asphyxia, adrenalin effects, and so on where the blood-pressure rises within four or five minutes, the hæmoglobin and corpuscular content perceptibly augment, conversely without exception in all conditions in which the arterial pressure falls the hæmoglobin value falls.

There can be little doubt but that these results can only be explained by the increased pressure forcing fluid out of the blood in the capillary districts into the tissue-spaces, and the passage of fluid back from the tissue-spaces into the blood when the pressure is lowered.

Is the blood-pressure raised at high levels ? If so, there is a complete explanation of the early polycythæmia which is a physiological fact related to this, and one which there is no need to account for by the hypothesis that an increase of hæmoglobin is an adjustment for the diminished tension of oxygen. The answer to this question varies, some observers finding an increase in the arterial pressure, while others find none. There is, however, uniform agreement that the pulse-rate is greater at high than low levels ; and this persists even after acclimatisation, though with this the arterial pressure is stated to be practically unaltered. All individuals, no matter what the extent of exercise, exhibit in response to this, respiratory and circulatory changes of the same order as at a low level, but in a greater degree. Since this is the case, the slightest exercise or even standing on one's feet would be followed by a rise of pressure exceeding that at the sea-level. We may believe that it is the rise in arterial pressure, which Scott's experiments have so clearly shown to affect the corpuscular and hæmoglobin content, that gives a rational explanation of the polycythæmia of high levels.

The relation of the pulse-rate to exercise is well known, but in 1909 Miss F. Buchanan discovered an absolutely unknown feature of this accelerated rate.

She was the first to show that in man the pulse quickens at the very commencement of voluntary exercise. The first diastolic period in the cardiac cycle is shortened at once. During the next few seconds acceleration of the pulse proceeds, becoming greater and reaching a steady level within two minutes, which is maintained so long as the muscles continue at work. Observations on seven different subjects show the extent of this change in the pulse-rate :—

| Resting. | After commencement of work. | | | | | |
|---|---|---|---|---|---|---|
| | Sec. 0.5 | Sec. 2.0 | Sec. 10.0 | Sec. 20.0 | Min. 1–2 | Min. 5–7 |
| 42 | 74 | 84 | 90 | 105 | 120 | 120–127 |
| 55 | 67 | 80 | 96 | 100 | 100 | 115 |
| 58 | 78 | 88 | 100 | 100 | 140 | 150 |
| 60 | 72 | 75 | 84 | 88 | 102 | 100 |
| 64 | 72 | 80 | 90 | 90 | 100 | 101 |
| 68 | 100 | 106 | 120 | 124 | 138 | 138 |
| 72 | 90 | 96 | 105 | 108 | 110 | 120 |

This immediate acceleration of the pulse in voluntary movement is due entirely to loss of the normal inhibitory effect of the vagus on the heart. Should the heart quicken from loss of vagal tone, this is shown by shortening of the diastolic period alone, the duration of the systole remains constant. Should the heart quicken from the augmentor effect of the sympathetic, then the time-relations of both systole and diastole are shortened. Since it has been shown by Krogh and Lindhard in 1913 that this initial quickening of the pulse is absent when the limbs are passively moved, we may conclude that the promptness with which the pulse accelerates at the commencement of voluntary exercise is due to impulses which spread from the motor areas of the cortex to the vagus centre. The initial acceleration is therefore purely psychical in origin, but doubtless other causes, such as an outflow of impulses via the accelerator nerves and the rise of temperature during exercise, contribute to the maintenance of an increased cardiac rate during exercise.

The prevailing conception of the capillary circulation among physiologists and pathologists appears to be that the capillaries are passive channels along which the blood is flowing continuously through all of them at rates which are determined by the state of contraction or dilatation of the pre-capillary arterioles. The accuracy of this view has recently been questioned by a number of observers. In two papers, "On the number and distribution of capillaries in muscles," and on "The supply of oxygen to the tissues and the regulation of the capillary circulation," August Krogh, of Copenhagen, has reviewed the existing experimental evidence and brought forward his own results, which show that the capillaries are not merely passively dilated by the blood-pressure, but that they constantly perform active changes in calibre. It cannot be assumed that the capillaries possess elasticity, and there is no evidence that this is the case. But the protoplasmic tube of living cells can conceivably actively dilate or constrict so as to obliterate its lumen ; in other words, react to stimuli which cause maximal contraction. On removal of the stimulus a relaxation to a mean position may occur, and would correspond to a state of capillary tone, and this feature would be a property of the capillaries themselves, and not one connected with any influence of a nervous system, such as is known to occur for the voluntary muscles, nor am I aware of any histological observations which would favour this idea.

It would seem that in the resting muscles most of the capillaries are in a state of contraction and closed to the passage of blood, but by throwing these into activity, or by gentle massage, a large number of capillaries are opened up. With cessation of these procedures the capillaries can be observed to close again. It is, of course, a matter of considerable difficulty to reach a high degree of accuracy by direct observation of the capillary circulation, but it seems certain that not only do channels which were invisible suddenly come into view, but the bore of individual capillaries is constantly changing, and this quite independently of the blood-pressure. On the assumption that the methods employed are reliable for deciding the variations in the capillary circulation of resting and active muscles, the following figures illustrate the differences. Stimulation of the sartorius shows 305 and 340 open capillaries per square millimetre respectively, the resting muscle showing 85, 96 and 100 per millimetre. The amount of blood which can be present in the capillaries of muscle is not less than 700 times the minimal quantity. The hyperæmic condition of any organ is due to a large number of capillaries being open and more or less dilated. In the anæmic condition most of the capillaries are closed, and most of those which remain open are more or less contracted. From the teleological point of view clearly the variations in the capillary bed of active muscles is related to the requirements of these organs for the supply of oxygen and removal of metabolites.

Among experiments which are inadequate to elicit the truth, even though these are carried out with the tonic of self-criticism, I have selected one which is given with much detail by Lister. In what was destined to be the last of his addresses he draws attention to the misleading views which even still exist as to the microscopical appearances of the initial stages of inflammation. Having repeated these, it is possible with some degree of assurance to hold views which are at variance with the current statements given in text-books, copied as these often are from one to another. To quote Lister's own words: " He[1] had never experimented on a perfectly healthy state of the circulation, but had described with great accuracy what could occur only under morbid conditions. For I afterwards learned that the normal temperature of man is deadly to the cold-blooded frog. That animal, which under ordinary conditions exhibits very remarkable persistence of vitality, even after somatic death, is killed by being held for about a quarter of an hour in the hand ; and if one of its hind feet be similarly warmed the blood-corpuscles will be found packed and stagnant in the vessels of the webs, as if mustard or any other powerful irritant had been applied to them. If on the other hand, in securing the frog for observation under the microscope, scrupulous care was taken to avoid needless exposure of the foot to the warmth of the hands, the threads for fixing the toes being held with long forceps, and each half of the knot tied separately, with a fair interval between them ; a state of the circulation was seen which is, I believe, even to this day rarely witnessed. The white corpuscles, instead

[1] *i.e.* Wharton Jones.

of trailing more or less sluggishly along the walls of the venous radicles—the normal condition, according to some modern text-books—move freely along among the red discs, there being diffused through a due proportion of liquor sanguinis, the vessels present a pallor which would surprise anyone who had seen only the ordinary demonstrations of the circulation."

The nature and behaviour of hæmoglobin has ever been a subject of fascination to physiologists, and if we consider that in a sense the complicated processes of the higher animals have been built up around this body, it is not a matter for wonder why this is so. No known chemical substance among the half-million carbon compounds known to chemists possesses the properties of hæmoglobin. We are ignorant of its mode of formation either in the embryo or the adult, but by this statement I do not mean that many theories as to its formation do not exist. It is well known that after severe loss of blood, for example during post-hemorrhagic anæmia, the corpuscular content is more rapidly regained than is the normal hæmoglobin content of the corpuscles so that the colour index of the corpuscles is reduced, the blood in this respect resembling cases of chlorosis. For my part I can think of no place where this diminution in hæmoglobin content for each corpuscle can ultimately be made good except in the medium in which the corpuscles float; moreover, it is now known that a many-sided exchange takes place between the constituents of the plasma and corpuscles.

Crystals of hæmochromogen—the reduced alkaline hæmatin of some text-books—are as easy to prepare as hæmin crystals. Beautiful rose-coloured prisms can be prepared, and photographs of these from different animals are shown upon the screen. I find that solutions of hæmochromogen may be prepared of a high degree of purity by the solution of hæmin crystals in hydrazin hydrate. With such solutions it is found that though hæmochromogen is the coloured iron-containing moiety of hæmoglobin, this substance in its relation to oxygen resembles hæmocyanin, the bluish copper-coloured pigment in the octopus and king-crabs. Both hæmochromogen and hæmocyanin behave not like hæmoglobin, but like calcium carbonate or reducible dye-stuffs like indigo or malachite green, which exist in an oxidised or reduced form. Just as hæmocyanin when oxidised is blue, so hæmochromogen when oxidised to hæmatin is brown; but these oxidised forms do not give off any oxygen to a vacuum nor dissociate under reduced oxygen pressure. Only chemical oxidising and reducing substances are active, and then these pigments are completely oxidised or completely reduced, features which sharply differentiate them from hæmoglobin.

J. A. Gardner and myself conclusively showed in 1906 that chloroform is carried not by the plasma but by the red corpuscles of the blood, though whether by the hæmoglobin or the stroma it is difficult to say. During the past five years I have attempted to ascertain the accuracy of the views which were independently held by Setchenow and Bohr on the function of hæmoglobin as a transporter of carbon dioxide in the blood. Can this gas be carried by this vehicle from the cells to the pulmonary capillaries? If this is the case, can hæmoglobin, apart from its accepted rôle as a transporter of oxygen, at the same time effect the carriage of carbon dioxide? This question involves several others, in particular the function of bicarbonate of sodium in the plasma and corpuscles. Considered by itself, bicarbonate cannot possibly lose any of its carbon dioxide at the lungs, for the pressure of this gas is so high in the alveoli of the lungs that any dissociation is out of the question. Solutions of sodium bicarbonate will not lose any gas at a pressure higher than 4 mm. of mercury, and that in the alveolar air is ten times this value. The general teaching is that proteins act as weak acids—they must be stronger than carbon dioxide—and effect a liberation of this gas from bicarbonate. The whole matter rests on the accuracy or inaccuracy of this idea, and the difficulties of proof in either direction are considerable. It is, however, certain that the regulation of the reaction of the blood is a function of bicarbonate of sodium.

Since a healthy disbelief in, or at any rate a suspension of judgment upon current theories maintains our interest in scientific work, and compels the repetition of old experiments and the devising of new ones, a brief account of my recent work on the relations which obtain between hæmoglobin and carbon dioxide may be alluded to. When pure, salt-free hæmoglobin crystals go into solution in water it is found that the capacity of such solutions to hold carbon dioxide augments as the proportion of hæmoglobin augments. This pigment, therefore, enables quantities of this gas to be retained, and in amounts enormously in excess of what water alone, or a solution of other proteins in water can hold. When the calculations are made in the case of salt-free blood with the assumption that the blood proteins can absorb carbon dioxide to an extent equal to that of hæmoglobin—though this is not the case—then the following table shows that even with this admission, the blood pigment absorbs large amounts of carbon dioxide gas :—

| Hb% in water. | Carbon dioxide absorbed by hæmoglobin in c.c. at 0° and 760 P. | 1 gram Hb absorbs. |
|---|---|---|
| 2.5 | 9.073 | 1.1 |
| 4.1 | 22.721 | 1.2 |
| 6.2 | 29.12 | 1.13 |
| 8.8 | 41.52 | 1.1 |
| 14.0 | 101.8 | 1.14 |
| 18.0 | 128.67 | 1.15 |

It is true that these figures do not prove that hæmoglobin actually does transport carbon dioxide in the body, but with the knowledge that the function of sodium bicarbonate does not appear to be that of a transporter of this gas these experiments have caused a renewed interest to be taken in this question. I have also demonstrated the essential fact that pure, unchanged solutions of hæmoglobin do not act in the sense of acids and bring about a liberation of carbon dioxide from bicarbonates, a view widely held and current in nearly all books.

The blood itself has always been a never-ending subject of research. "Oh yrkesome knowledge! Oh science full of molestation; that wasteth for us the sweetest houres of life," is a reflection by Montaigne well adapted to the minds of those in the past and present time who have attempted a study of this mysterious fluid. "Blut ist ein ganz besonderer Saft," says Mephistopheles, and so it is for us all. The proteins of the blood, regarded at one time as the source whence the cells of our bodies draw their nitrogen requirement, are now known not to serve the cells in this manner; these obtain nitrogen from the amino-acid content of the plasma, the proteins playing a humbler part in preventing a too rapid exit of water from the capillaries. As to the reaction of the blood, knowledge is becoming much more definite, and it is permissible to believe that the condition to which Naunyn in 1906 gave the name of acidosis will be restricted to those abnormal metabolic conditions in which $\beta$-hydroxybutyric acid and allied bodies are formed. The confused ideas of acidosis and experimental acid intoxication do not sufficiently differentiate these two forms of alteration in the character of the blood. In the sense in which Naunyn employed this term acidosis does not necessarily produce any effect at all on the acid-base balance of the body, and the bodies which cause acidosis, indicating incomplete oxidation of fats or amino-acids, are significant of specific errors of metabolism, while acid-intoxications do actually reduce the bicarbonate reserve of the blood.

W. M. Bayliss and T. R. Parsons have both, though by different methods, shown how remarkably constant the reaction of our blood remains throughout life, and this is maintained by an adjustment of the loss of carbon dioxide by the lungs and of salts by the kidney. Strengths of acidity are expressed in various forms of notation, which indicate the hydrion concentration of acids in solution. The range of this compatible with life is exceedingly minute. Many common indicators used by chemists, such as phenolphthalein, methyl-orange and methyl-red, change in tint at hydrogen-ion concentrations far outside the range of the blood. Neutral red, and the recent indicators brom-cresol purple and phenol red suggested for use by the Medical Research Committee, do change quite sharply between the values of $10^{-7}$ and $10^{-8}$, the limits of hydrogen-ion concentration which are compatible with life according to the work of L. J. Henderson.

An old remark of Michael Foster's comes into one's mind n reflecting on the many-sided nature of the sciences of physiology and pathology: "In their methods, in their aims they are identical, and indeed a definitely united subject. As there is not a science of meteorology of good weather and another of bad weather, so there is not a separate science for the processes of health and another for those of disease."

